# Proteomic insight into the pathogenesis of CAPN5-vitreoretinopathy

**DOI:** 10.1038/s41598-019-44031-7

**Published:** 2019-05-20

**Authors:** Gabriel Velez, Jing Yang, Angela S. Li, Stephen H. Tsang, Alexander G. Bassuk, Vinit B. Mahajan

**Affiliations:** 1 Omics Laboratory, Stanford University, Palo Alto, CA USA; 2 Department of Ophthalmology, Byers Eye Institute, Stanford University, Palo Alto, CA USA; 3 Medical Scientist Training Program, University of Iowa, Iowa City, IA USA; 4 Palo Alto Veterans Administration, Palo Alto, CA USA; 5 Jonas Children’s Vision Care, and Bernard & Shirlee Brown Glaucoma Laboratory, Columbia Stem Cell Initiative, Departments of Ophthalmology, Pathology & Cell Biology, Institute of Human Nutrition, Columbia University, New York, NY USA; 6 Department of Pathology & Cell Biology, College of Physicians & Surgeons, Columbia University, New York, NY USA; 7 Department of Pediatrics, University of Iowa, Iowa City, IA USA

**Keywords:** Proteomics, Biomarkers, Eye diseases

## Abstract

CAPN5 Neovascular Inflammatory Vitreoretinopathy (CAPN5-NIV; OMIM 193235) is a poorly-understood rare, progressive inflammatory intraocular disease with limited therapeutic options. To profile disease effector proteins in CAPN5-NIV patient vitreous, liquid vitreous biopsies were collected from two groups: eyes from control subjects (n = 4) with idiopathic macular holes (IMH) and eyes from test subjects (n = 12) with different stages of CAPN5-NIV. Samples were analyzed by liquid chromatography-tandem mass spectrometry (LC-MS/MS). Protein expression changes were evaluated by principal component analysis, 1-way ANOVA (significant p-value < 0.05), hierarchical clustering, gene ontology, and pathway representation. There were 216 differentially-expressed proteins (between CAPN5-NIV and control vitreous), including those unique to and abundant in each clinical stage. Gene ontology analysis revealed decreased synaptic signaling proteins in CAPN5-NIV vitreous compared to controls. Pathway analysis revealed that inflammatory mediators of the acute phase response and the complement cascade were highly-represented. The CAPN5-NIV vitreous proteome displayed characteristic enrichment of proteins and pathways previously-associated with non-infectious posterior uveitis, rhegmatogenous retinal detachment (RRD), age-related macular degeneration (AMD), proliferative diabetic retinopathy (PDR), and proliferative vitreoretinopathy (PVR). This study expands our knowledge of affected molecular pathways in CAPN5-NIV using unbiased, shotgun proteomic analysis rather than targeted detection platforms. The high-levels and representation of acute phase response proteins suggests a functional role for the innate immune system in CAPN5-NIV pathogenesis.

## Introduction

**Figure 1:**
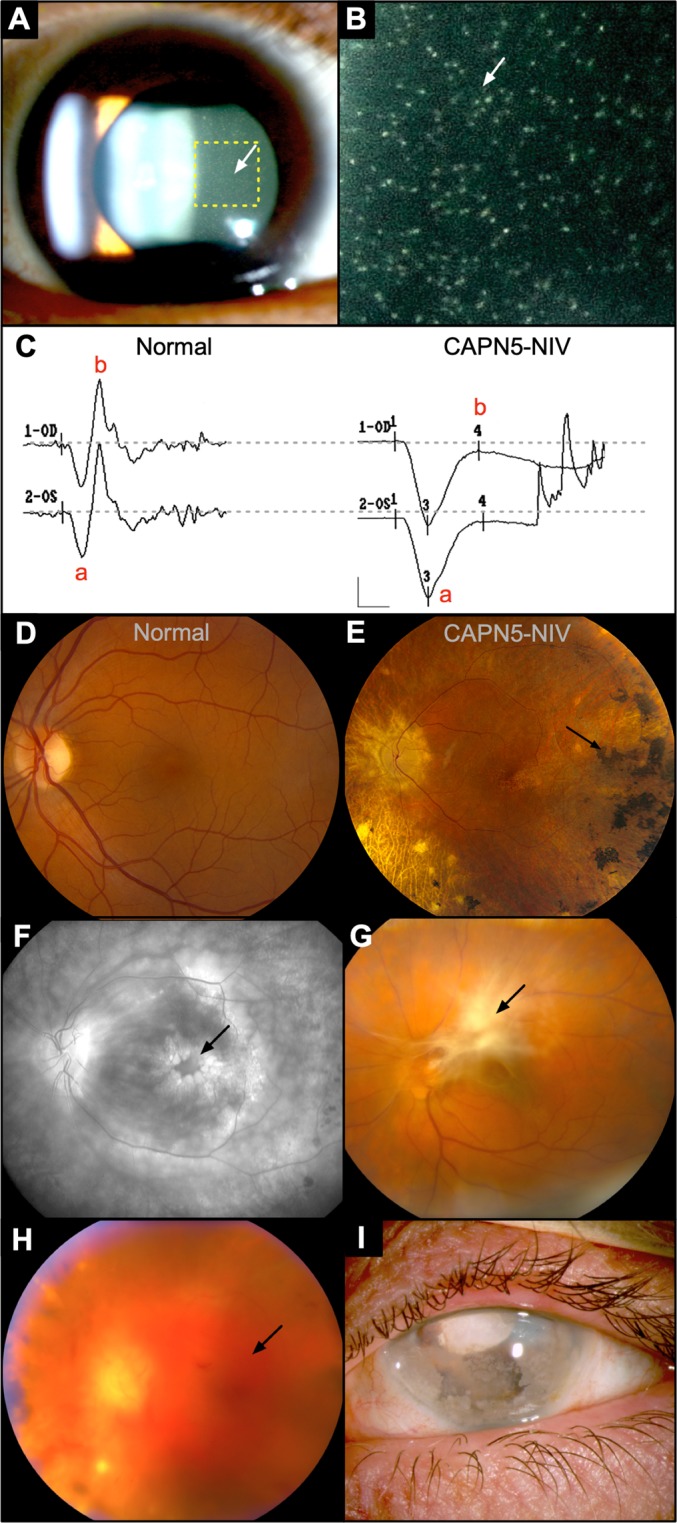
Clinical CAPN5-NIV phenotype: (**A**,**B**) Clusters of autoimmune reactive leukocytes in the vitreous cavity (inset, arrows). (**C**) Electroretinography reveals early synaptic signaling defects in CAPN5-NIV patients, detected as loss of the b-wave. (**D**) Fundus image of the normal retina. (**E**) Fundus image of CAPN5-NIV retina showing pigmentary degeneration (arrow). (**F**) Fluorescein angiography reveals cystoid macular edema at the fovea (arrow), a consequence of intraocular inflammation. (**G**) Intraocular fibrosis and pre-retinal scar tissue formation (arrow). (**H**) Vitreous hemorrhage (arrow) caused by retinal neovascularization. (**I**) Phthisis bulbi and involution of eye tissues at end-stage CAPN5-NIV disease. Images courtesy of Mahajan, *et al*. (2012).

CAPN5 Neovascular Inflammatory Vitreoretinopathy (CAPN5-NIV; OMIM 193235) is a rare, progressive inflammatory intraocular disease caused by mutations in the *CAPN5* gene^1^. Before culminating in blindness, CAPN5-NIV disease progresses in a series of pathological stages, characterized by synaptic signaling defects (loss of b-wave on electroretinogram), inflammatory cell infiltration, neovascularization, and intraocular fibrosis (Fig. 1). These 5 stages each mimic common eye diseases that together account for a significant fraction of visual morbidity and blindness (e.g. uveitis, retinitis pigmentosa, proliferative diabetic retinopathy, and proliferative vitreoretinopathy)^2^. The *CAPN5* gene encodes for calpain-5, a regulatory protease expressed in photoreceptors that modulates the biological function of its proteolytic targets^3,4^. CAPN5-NIV- mutations cause a gain-of-function, have been shown to hyper-activate the protease^5–7^. Although several CAPN5-NIV-causing mutations have been identified, it is not known how a hyperactive protease leads to uveitis^1,7,8^. Since the underlying mechanisms of CAPN5-NIV are poorly understood, these patients are left with few treatment options and fail conventional immunosuppressive therapy, such as oral corticosteroids and infliximab (anti-TNF-α)^2^.

To identify therapies for NIV, our group has previously taken a drug repositioning approach to treating CAPN5-NIV patients. Drug repositioning applies approved drugs towards new indications, which often are rare diseases with few therapeutic options^2^. To identify candidate drugs for repositioning, we have used a personalized proteomics approach^2,9^. Liquid vitreous biopsies from CAPN5-NIV patients undergoing surgery were analyzed by a multiplex ELISA array that simultaneously measured levels of 200 human cytokines. The levels of these cytokines were subsequently analyzed by heatmap clustering and pathway analysis to identify proteins that may be targeted by available drugs. This analysis showed that TNF-α levels were normal, explaining why infliximab therapy failed in these patients. It was further revealed that the CAPN5-NIV vitreous contained abundant levels of vascular endothelial growth factor (VEGF), T-cell proliferative markers, and interleukin-6 (IL-6). Based on this, we repositioned bevacizumab (anti-VEGF monoclonal antibody), intravitreal methotrexate (T-cell inhibitor), and tocilizumab (anti-IL-6 monoclonal antibody) and successfully mitigated neovascularization, inflammatory cell infiltration, and persistent fibrosis in these patients^2^.

Despite the success of our past personalized proteomics studies for CAPN5-NIV, the original analysis was limited to only 200 cytokines. Since different proteomic platforms can give different results, we sought to obtain a more global view of the CAPN5-NIV vitreous proteome at each stage. Liquid chromatography-tandem mass spectrometry (LC-MS/MS) is a powerful, unbiased analytical technique that ionizes molecular species in complex biochemical mixtures and sorts ions based on their mass-to-charge ratio (*m/z*)^10,11^. This technique is used in a “shotgun” approach to catalog and quantify the hundreds to thousands of proteins in a biological sample, which is often performed in tandem with liquid chromatography to separate peptides before they are ionized^10^. We have utilized LC-MS/MS techniques to catalogue proteins in the normal human retina, vitreous, and retinal pigmented epithelium (RPE)-choroid complex^12–14^. Here, we describe a similar shotgun proteomics study of CAPN5-NIV vitreous, using LC-MS/MS, and perform bioinformatic analysis to detect stage-specific changes and identify potential drug targets for further drug repositioning.

## Results

### Differential protein expression

**Figure 2:**
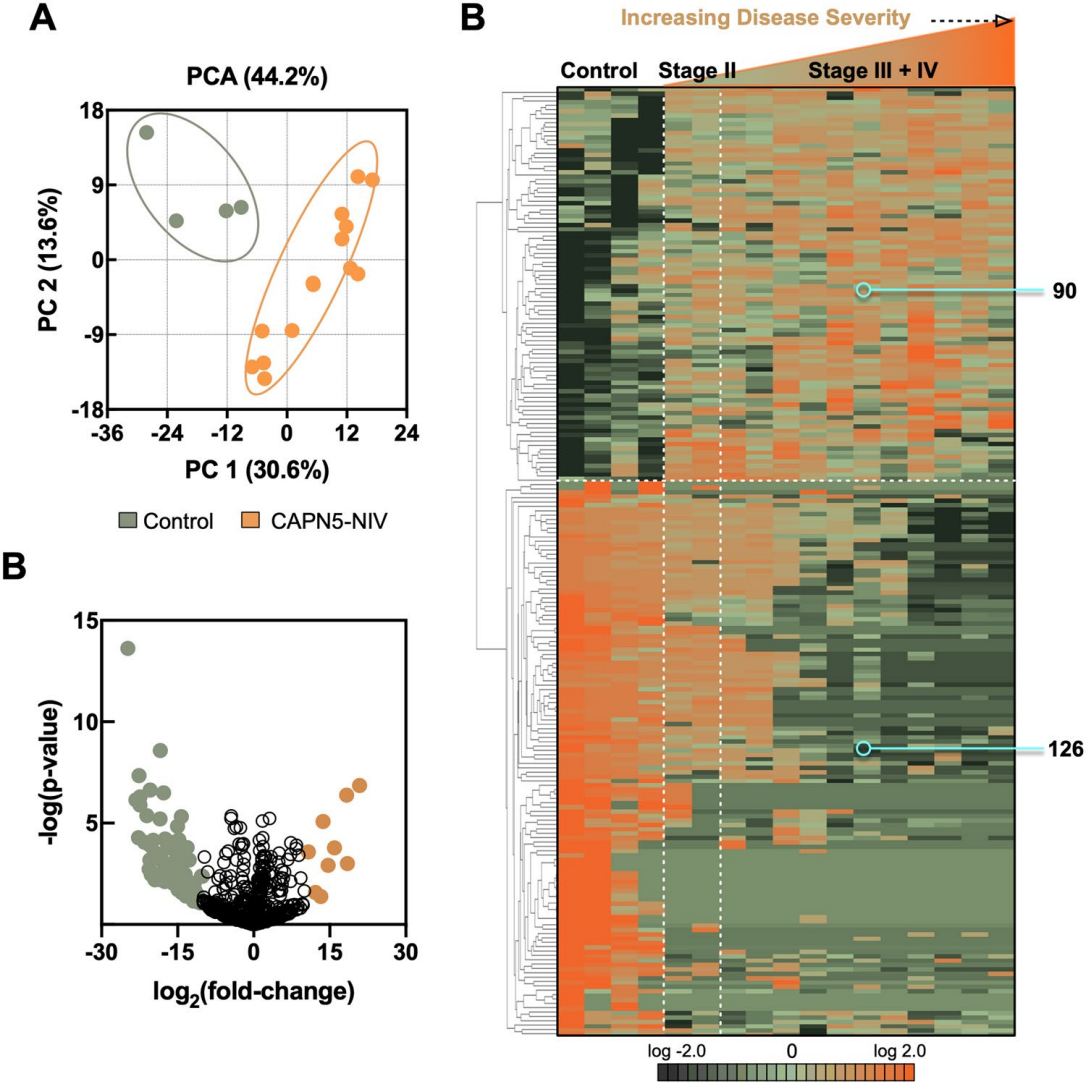
Differentially-expressed proteins reveal differences between CAPN5-NIV cases and controls: (**A**) Principal component analysis (PCA) of the proteomics data. The score plot of PC1 and PC2 shows separation between CAPN5-NIV cases (orange) and IMH controls (green) based on protein intensities that were significantly different between the two groups. (**B**) Protein intensities were analyzed by 1-way ANOVA. Results are represented as a volcano plot. The horizontal axis (x-axis) displays the log2 fold-change value (CAPN5-NIV vs. controls) and the vertical axis (y-axis) displays the noise-adjusted signal as the -log10 (p-value) from the 1-way ANOVA analysis. (**C**) Protein intensities were analyzed by hierarchical clustering. A total of 216 proteins were differentially-expressed among control (IMH) and CAPN5-NIV samples (90 upregulated proteins in CAPN5-NIV samples and 126 downregulated proteins; p < 0.05). Results are represented as a heatmap and display protein expression levels on a logarithmic scale. Orange indicates high expression while dark green/black indicates low or no expression.

Vitreous samples from 8 CAPN5-NIV patients (12 eyes) and 4 idiopathic macular hole (IMH) controls underwent trypsinization and liquid chromatography before analysis by tandem mass spectrometry (Table 1; Supplemental Fig. 1). In control IMH vitreous, we identified 373 ± 70 unique proteins (mean ± SD; n = 4). There were 428 ± 16 unique proteins in Stage II CAPN5-NIV (n = 2), 344 ± 55 in Stage III CAPN5-NIV (n = 5), and 392 ± 56 (n = 5) in Stage IV CAPN5-NIV (Table 1). The most abundant proteins shared among all samples were: serotransferrin (TF), alpha-1-antitrypsin (SERPINA1), and apolipoprotein A-I (APOA1). These proteins were also abundant in Stage II vitreous. The most abundant proteins in control samples were immunoglobulin heavy constant gamma 1 (IGHG1) and transthyretin (TTR). Immunoglobulin heavy constant gamma 1 (IGHG1), complement C3 (C3), and alpha-1-antichymotrypsin (SERPINA3) were the most abundant proteins in CAPN5-NIV samples. Proteomics data were then compared using principal component analysis (PCA). The score plot of PC1 and PC2 showed separation between the 12 CAPN5-NIV cases and 4 IMH controls based on protein intensities that were significantly different between the two groups (Fig. 2A). Protein intensities were further analyzed using 1-way ANOVA and hierarchical clustering. A total of 216 proteins were differentially-expressed among control and CAPN5-NIV samples (90 upregulated proteins in CAPN5-NIV samples and 126 downregulated proteins; p < 0.05; Fig. 2B,C).

**Table 1:** CAPN5-NIV patient demographics.

Patient	Sex	Age^*^	Eye	Surgical Indication	Diagnosis	Unique Proteins
**CAPN5-NIV Samples**						
1	F	69	OS	Vitrectomy	Stage 3 NIV	382
2^†^	F	31	OD	Vitrectomy, membrane peel, Retisert	Stage 3 NIV	270
		31	OS	Vitrectomy	Stage 2 NIV	407
3^†^	F	42	OD	Vitrectomy, Retisert	Stage 3 NIV	290
		38	OS	Vitrectomy	Stage 2 NIV	429
4^†^	M	30	OD	Retisert removal	Stage 3 NIV	420
5^†^	F	69	OD	Vitrectomy, Retisert	Stage 4 NIV	372
		70	OS	Vitrectomy	Stage 4 NIV	441
6	F	68	OD	Retisert	Stage 4 NIV	444
		70	OD	Vitrectomy, Retisert exchange	Stage 4 NIV	317
7	F	62	OS	Vitrectomy, Retisert	Stage 4 NIV	389
8	F	31	OD	Vitrectomy	Stage 3 NIV	368
**Control Samples**						
9	M	66	OD	Vitrectomy	Macular Hole	421
10	F	56	OD	Vitrectomy	Macular Hole	370
11	F	57	OD	Vitrectomy	Macular Hole	310
12	M	68	OD	Vitrectomy	Macular Hole	471

^*^Age at the time of surgery.

^†^From same pedigree.

### Gene ontology analysis

**Figure 3:**
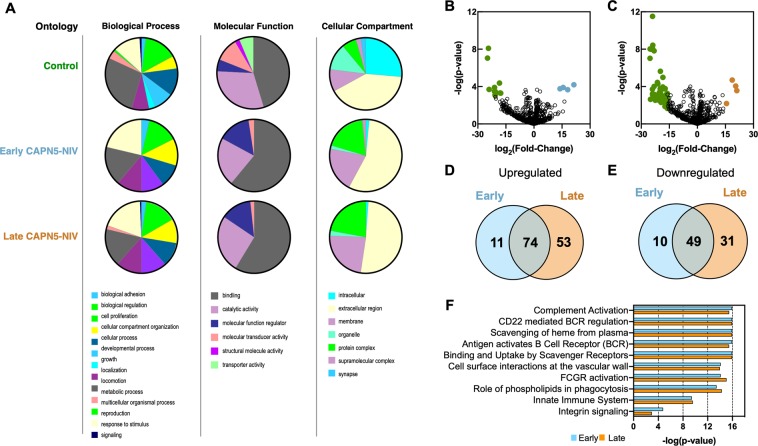
Gene ontology and pathway analysis reveals differences among early and late CAPN5-NIV: (**A**) Differentially**-**expressed proteins from early and late CAPN5-NIV compared to controls. Gene ontology analysis categorized each protein group by biological process, molecular function, and cellular compartment. (**B**,**C**) Differentially**-**expressed proteins from early CAPN5-NIV compared to controls. Results are represented as a volcano plot. The horizontal axis (x-axis) displays the log2 fold-change value (early CAPN5-NIV vs. controls) and the vertical axis (y-axis) displays the noise-adjusted signal as the -log10 (p-value) from the 1-way ANOVA analysis. Proteins with log2 fold-changes greater than 15 (upregulated) are colored cyan (early) and orange (late) while proteins with log2 fold-changes lower than −15 are represented in green (downregulated). (**D**) Comparative analysis of upregulated proteins (compared to controls) using Venn diagrams. A total of 74 upregulated proteins are shared among the two stages compared to controls (p < 0.05). (**E**) Comparative analysis of downregulated proteins using Venn diagrams. A total of 49 downregulated proteins are shared among the two stages compared to controls (p < 0.05). (**F**) Top ten pathways represented in CAPN5-NIV. Pathways are ranked by their −log (p-value) obtained from the right-tailed Fisher’s Exact Test.

To obtain a global view of the protein classifications represented in CAPN5-NIV vitreous, a gene ontology (GO) analysis was performed. Lists of differentially-expressed proteins between CAPN5-NIV and controls were classified by their respective biological process, molecular function, and cellular compartment (Fig. 3A). When proteins were categorized by cellular compartment, we observed a significant fraction of proteins localized to the synapse in controls that decreased in CAPN5-NIV vitreous. Among these proteins were: neurexin-2 (NRXN2), glutamate receptor 4 (GluR4), neurofascin (NFASC), neuronal growth regulator 1 (NREG1), calsyntenin-1 (CLSTN1), and oligodendrocyte-myelin glycoprotein (OMG). Patients in the early stages of CAPN5-NIV disease display altered synaptic signaling characterized by an early decrease in the b-wave amplitude on scotopic flash electroretinogram (rod photoreceptors)^4,15^. NRXN2 is a neuronal cell adhesion protein that is involved in synapse formation and synaptic regulation in the retina^16^. Loss of neurexin has been shown to significantly impair visual function by decreasing rhodopsin levels^16^. GluR4 is a metabotropic glutamate receptor expressed in the rod bipolar cells of the mammalian retina^17,18^. The loss of these synaptic proteins may contribute to the synaptic signaling defect seen in early CAPN5-NIV disease. However, our proteomic analysis could not differentiate which retinal cells are affected by loss of these proteins in CAPN5-NIV.

### Stage-specific protein expression

Each CAPN5-NIV stage is characterized by different clinical phenotypes and pathological processes, suggesting that they may contain distinct proteins. We therefore sought to identify stage-specific protein signatures that may correlate to these clinical features. First, we compared early CAPN5-NIV (Stages II and III) vitreous to controls (Fig. 3B). A total of 144 proteins were differentially-expressed among control and early CAPN5-NIV samples (86 upregulated proteins in CAPN5-NIV samples and 58 downregulated proteins; p < 0.05; Fig. 3A). Among the significantly upregulated proteins were scavenger receptor cysteine-rich type 1 protein M130 (CD163) and plastin-2 (LCP1). CD163 is a macrophage-specific receptor that indicates the presence M2 macrophages. M2 “repair” macrophages are required for vascular stability and produce VEGF. Previous studies have shown that CD163 production by human macrophages drive cytokine secretion in favor of an anti-inflammatory phenotype^19^. LCP1 plays a role in activating T-cells by facilitating transport of CD69 and CD25^20^. Among the significantly-downregulated proteins were several proteins involved in retinal function: retinoschisin (RS1), and opticin (OPTC). RS1 is involved in retinal cell adhesion and is required structure and function in the retina. Loss of RS1 function is most-notably associated with retinoschisis, an inherited vitreoretinal dystrophy associated with vitreous hemorrhage, retinal detachment, and neovascular glaucoma^21^. Opticin is an extracellular matrix glycoprotein associated with the collagen-integrin matrix in the vitreous. It has been previously shown to display anti-angiogenic activity^22^. Angiogenesis and vitreous hemorrhage are key features of CAPN5-NIV disease and downregulation of opticin may contribute to the increased retinal neovascularization seen in these patients^1,2^. There were proteins with known antioxidant activity present in controls that were absent in CAPN5-NIV vitreous: superoxide dismutase (SOD1 and SOD3), peroxiredoxin (PRDX2 and PRDX6), catalase (CAT), clusterin, and glutathione peroxidase 3 (GPX3). Clusterin is an antioxidant protein involved in the removal of cellular debris and has been implicated in reducing the breakdown of the blood-retinal barrier^23^. Past proteomic studies have shown clusterin levels to be reduced in the vitreous of diabetic retinopathy patients^23^. CAPN5-NIV patients similarly display breakdown of the blood retina barrier leading to vascular leakage of protein into the vitreous^2^. Taken together, these results indicate that early CAPN5-NIV stages are characterized by reduced defense against oxidative stress.

Next, we compared late CAPN5-NIV (Stage IV) vitreous to controls (Fig. 3B). A total of 206 proteins were differentially-expressed among control and late CAPN5-NIV samples (79 upregulated proteins in CAPN5-NIV samples and 127 downregulated proteins; p < 0.05; Fig. 3C). Among the significantly-upregulated proteins were those involved in the acute phase response and coagulation cascade: complement components (C1R, C6, C7, C8, and C9), prothrombin, antithrombin 3 (AT3), hemopexin (HPX), and carboxypeptidase B2 (CPB2). Acute phase proteins are a class of proteins whose plasma levels rise or fall dramatically in response to an injury or acute inflammatory event^24,25^. Many of the acute phase proteins fall into several overlapping functional categories, including complement signaling, coagulation and fibrinolysis, and inflammatory mediators^25^. Although most acute phase proteins are produced and secreted by hepatocytes, we have previously reported their expression in the normal human vitreous and RPE-choroid complex^13,14^. Several crystallin proteins were also upregulated (CRYAA, CRYAB, CRYBB1). Crystallins are soluble proteins found in the lens and cornea and were previously shown to have angiogenic properties^26^. Patients with proliferative diabetic retinopathy display increased vitreous levels of CRYAB, which correlate to increased levels of VEGF^27^. Consistent with this observation, CAPN5-NIV patients were previously found to have elevated vitreous VEGF levels and intravitreal injection of bevacizumab (anti-VEGF) suppresses neovascularization and clears vitreous hemorrhages^2^. Among the downregulated proteins was versican (VCAN/CSPG2). Versican is a chondroitin sulfate proteoglycan involved in maintaining the physiologic structure of the vitreous. Interestingly, loss of versican expression and function is implicated in vitreoretinal degeneration (versican vitreoretinopathy; OMIM: 118661)^28^. A comparative analysis revealed 49 upregulated proteins and 74 downregulated proteins were shared among the three stages, suggesting that different stages of CAPN5-NIV may share similar pathways and classifications of proteins (Fig. 3D,E).

### Pathway analysis

**Figure 4:**
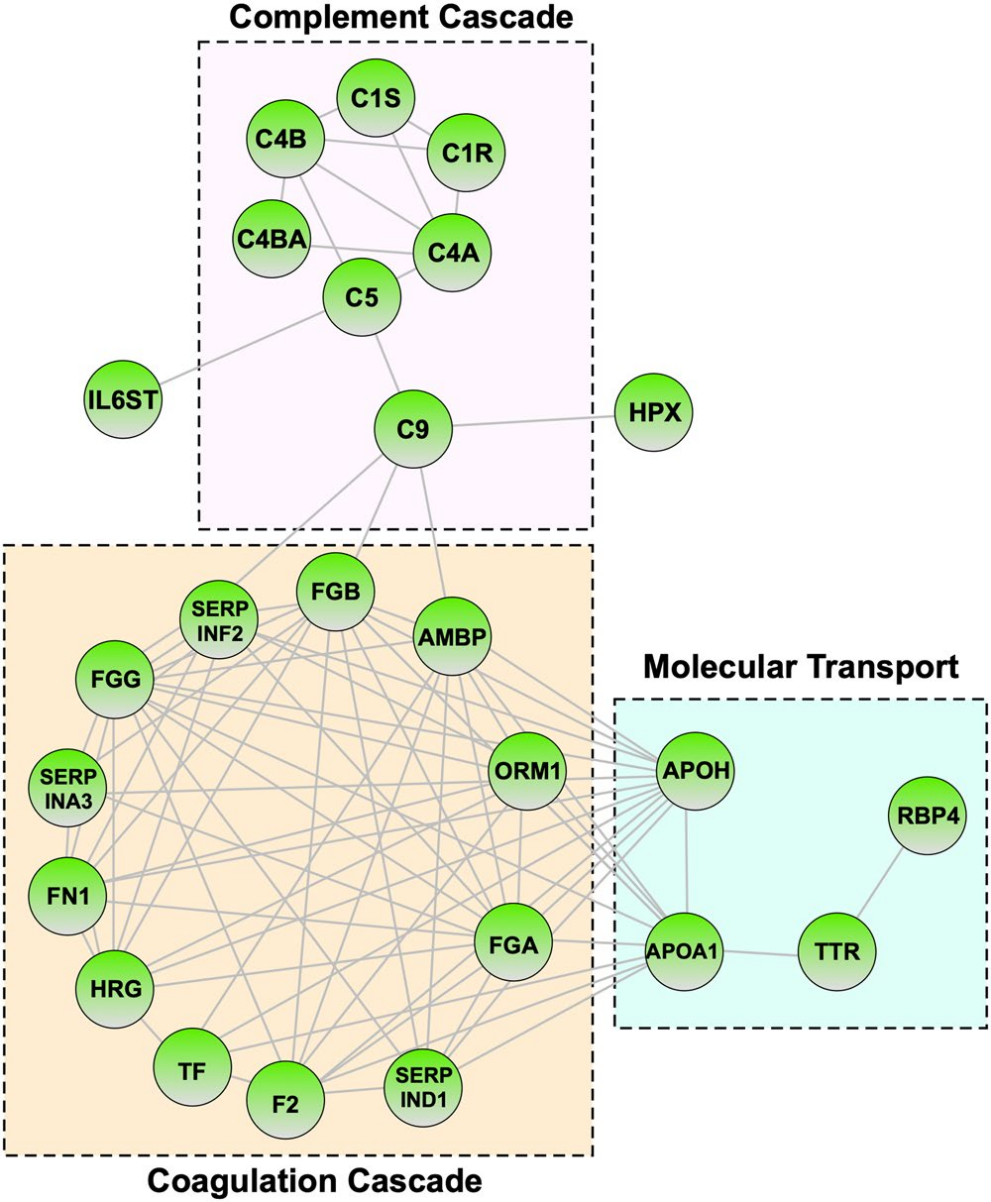
Acute phase response network: The acute phase response was the most-significantly represented pathway common to all CAPN5-NIV stages. There were 25 acute phase response proteins elevated in CAPN5-NIV. Results are displayed as a protein interaction network with proteins (nodes) represented as circles and connected by lines representing predicted or experimentally-confirmed interactions (edges). Nodes are highlighted by their respective molecular pathway or function: complement cascade, coagulation cascade, and molecular transport.

To further classify differentially-expressed proteins in CAPN5-NIV vitreous, we performed pathway analysis, which identifies groups of functionally-linked proteins. The top represented pathways in CAPN5-NIV were: complement activation, CD22 mediated B-cell receptor (BCR) regulation, scavenging of heme from plasma, BCR activation by antigens, binding and uptake of ligands by scavenger receptors, cell surface interactions at the vascular wall, FCGR activation, role of phospholipids in phagocytosis, innate immune system, and integrin signaling (Fig. 3F; Supplemental Tables 1–2). The high representation of coagulation cascade members may be due to the presence of blood in the vitreous during collection. Several of the patients underwent surgery for vitreous hemorrhage. The most significantly represented pathways were associated with the acute phase response. Increased levels of acute phase response proteins are a common feature of neuroinflammation, and complement signaling has been implicated in degenerative eye diseases like age-related macular degeneration (AMD)^29,30^. We identified a total of 25 interaction acute phase proteins that were significantly upregulated in CAPN5-NIV vitreous, suggesting that innate immune elements play a role in the pathogenesis of CAPN5-NIV (Fig. 4).

## Discussion

CAPN5-NIV is a poorly-understood inflammatory disease with few therapeutic options. Our data demonstrate significant molecular differences between CAPN5-NIV stages that may provide insight into the molecular mechanisms underlying the disease. Pathway analysis confirmed molecules and pathways previously identified in CAPN5-NIV vitreous (e.g. angiogenesis, IL-6 signaling, and T-cell signaling pathways)^2^. These findings support the current analytical approach. By using an unbiased proteomic approach rather than targeted proteomics, we identified affected new molecular pathways that were not previously associated with CAPN5-NIV, including acute phase response signaling, the complement cascade, oxidative stress, and synaptic transmission. Furthermore, the CAPN5-NIV vitreous proteome displayed characteristic enrichment of proteins and pathways previously-associated with non-infectious posterior uveitis, rhegmatogenous retinal detachment (RRD), age-related macular degeneration (AMD), proliferative diabetic retinopathy (PDR), and proliferative vitreoretinopathy (PVR; Tables 2 and 3).

**Table 2:** Commonly-enriched proteins between CAPN5-NIV and other vitreoretinal diseases: Upregulated proteins in CAPN5-NIV vitreous (compared to controls) that have been previously-identified in proteomic studies of other vitreoretinal diseases. AMD, age-related macular degeneration; PDR, proliferative diabetic retinopathy; PVR, proliferative vitreoretinopathy; RRD, rhegmatogenous retinal detachment.

Protein	CAPN5-NIV	AMD	PDR	PVR	RRD	Uveitis
Interleukin-6 (IL-6)	Elevated^2^			Elevated^51^		Elevated^52^
Vascular endothelial growth factor (VEGF)	Elevated^2^	Elevated^53^	Elevated^54^	Elevated^51^		
Platelet-derived growth factor B (PDGFB)	Elevated^2^		Elevated^55^			
Scavenger receptor cysteine-rich type 1 protein M130 (CD163)	Elevated					
Lymphocyte cytosolic protein 1 (LCP1)	Elevated					
Complement component C1R	Elevated	Elevated^31^			Elevated^56^	
Complement component C6	Elevated	Elevated^31^				
Complement component C7	Elevated	Elevated^31^	Elevated^57^			
Complement component C8	Elevated		Elevated^57^		Elevated^56^	
Complement component C9	Elevated	Elevated^31^	Elevated^57^	Elevated^58^	Elevated^56^	
Prothrombin (F2)	Elevated	Elevated^31^				
Anti-thrombin 3 (AT3)	Elevated					
Hemopexin (HPX)	Elevated			Elevated^58^		
Carboxypeptidase 2 (CPB2)	Elevated	Elevated^31^				

Superscript numbers denote the corresponding references.

**Table 3:** Commonly-enriched pathways between CAPN5-NIV and other vitreoretinal diseases: Differentially-expressed pathways in CAPN5-NIV vitreous (compared to controls) that have been previously-identified in proteomic studies of other vitreoretinal diseases. AMD, age-related macular degeneration; PDR, proliferative diabetic retinopathy; PVR, proliferative vitreoretinopathy; RRD, rhegmatogenous retinal detachment.

Pathway	CAPN5-NIV	AMD	PDR	PVR	RRD	Uveitis
Acute phase response signaling	Enriched					
Complement cascade	Enriched	Enriched^31^	Enriched^57^	Enriched^58^	Enriched^56^	
Oxidative stress defense	Downregulated	Enriched^31^	Downregulated^57^	Downregulated^58^		
Synaptic transmission	Downregulated				Enriched^56^	
VEGF and PDGF signaling	Enriched^2^	Enriched^53^	Enriched^54,55^	Enriched^51^		Enriched^9^
mTOR and PI3K signaling	Enriched^2^			Enriched^51^		

Superscript numbers denote the corresponding references.

**Figure 5:**
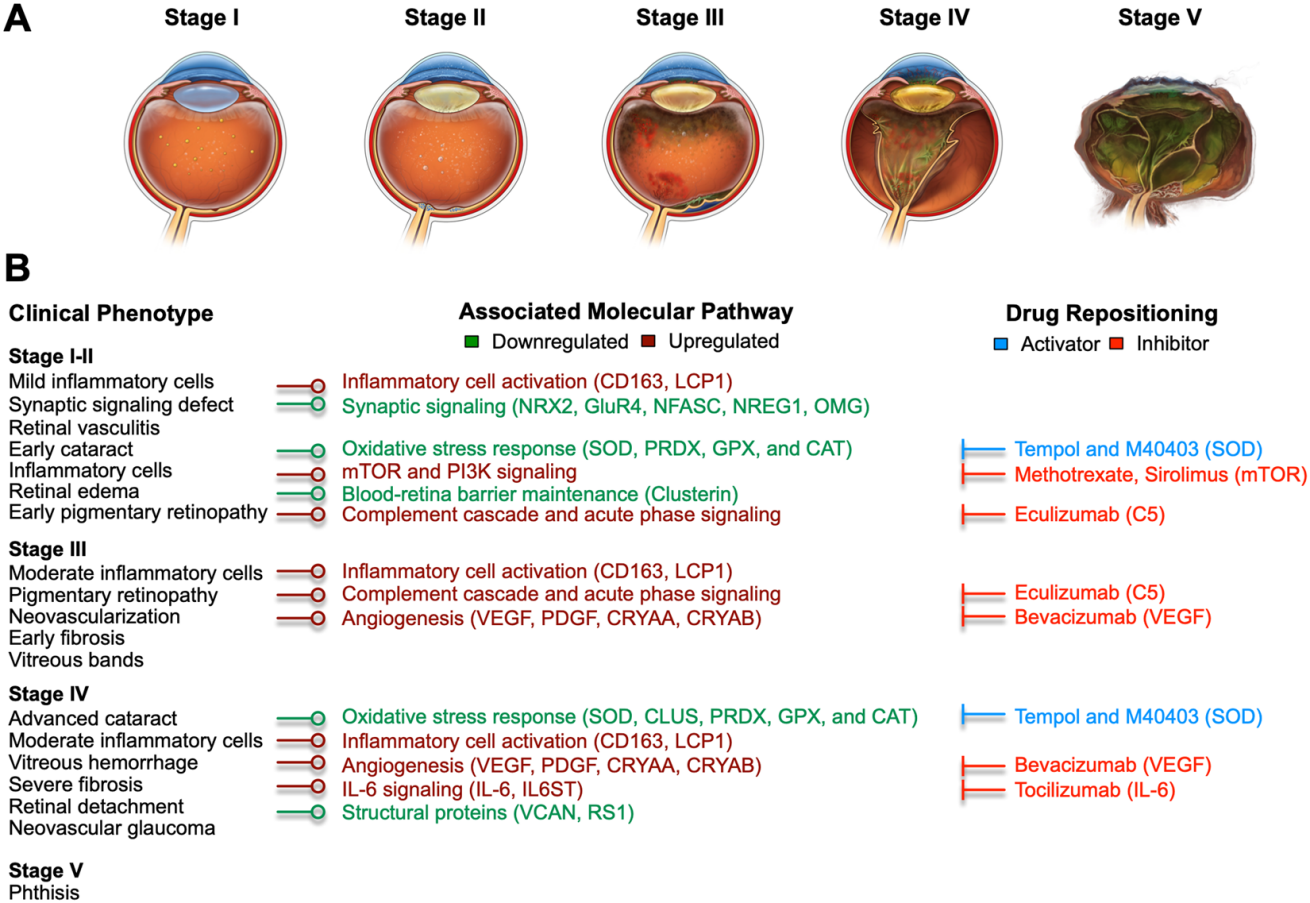
A CAPN5 vitreoretinopathy disease model for therapeutic testing: (**A**) Illustrations highlighting the clinical phenotype at each CAPN5-NIV stage. Graphical illustrations by Alton Szeto and Vinit Mahajan. Permission to publish granted by original artist. (**B**) A constructed disease model highlighting the molecular phenotype with associated proteins and correlation to clinical phenotype of CAPN5-NIV. Potential therapeutics which have already been approved or are in trial for use in other diseases are represented based on differentially-expressed proteins and pathways.

With insights into elevated proteins and pathways, we considered FDA-approved drugs or drugs that are currently in clinical trials that could be repositioned for CAPN5-NIV (Fig. 5A,B). For instance, the decrease in antioxidant proteins in later CAPN5-NIV stages may represent a reduced defense against reactive oxygen species (ROS) in later CAPN5-NIV stages. Oxidative stress is associated with photoreceptor damage in several retinal diseases including PDR, retinitis pigmentosa (RP), and AMD and different retinal regions display varying susceptibility to damage by ROS^12^. Past proteomic studies have showed enrichment for proteins involved in oxidative stress in AMD vitreous^31^. In our current study, we observed decreased levels of superoxide dismutase enzymes (SOD1 and SOD3) in CAPN5-NIV vitreous. SOD3 has been shown to regulate oxidative stress at the vitreoretinal interface and loss of *Sod3* in mice display inner retina signaling abnormalities^32^. Compounds that activate antioxidant proteins may be beneficial in treating or preventing photoreceptor damage that results from chronic oxidative stress. Several SOD-mimetic compounds (e.g. M40403 and tempol), for example, have been demonstrated to be protective in numerous animal models of acute and chronic inflammation, chemotoxicity, reperfusion injury, and shock^33–36^. These compounds may be administered early in CAPN5-NIV disease to prevent accumulation of damaging ROS.

The high levels and number of acute phase proteins suggests a potential important role for the innate immune system in CAPN5-NIV pathogenesis. Our previous proteomic studies linked several cytokine-signaling proteins and pathways involved in the adaptive immune response to CAPN5-NIV (e.g. mTOR and PI3K signaling pathways), although innate immune pathways were not detected due to the targeted nature of our analysis^2^. Innate immune elements have been previously detected in non-diseased eye tissues (e.g. anterior chamber, vitreous, RPE-choroid) and are implicated in a number of neurodegenerative diseases, such as AMD and RP^25,37,38^. Notably, there was higher representation of acute phase signaling in late CAPN5-NIV (Figs 3F; 4). This increased acute phase response representation may result from non-specific innate immune activation in response to progressive photoreceptor degeneration. Alternatively, hyperactive CAPN5 activity may cause tissue injury that triggers non-specific activation of these innate immune elements. There is some precedent for this as elevated calpain activity is associated with a wide range of disease, including retinal degeneration and neuronal injury^39–42^. Aberrant proteolysis of retinal CAPN5 substrates by a hyperactive protease may similarly lead to exposure of peptide epitopes that trigger the autoinflammatory response seen in CAPN5-NIV. Further research, however, is required to elucidate the interplay between the innate and adaptive immune system in CAPN5-NIV pathogenesis.

Our current study has several limitations. Most notably is the limited sample size, especially for Stage II CAPN5-NIV (Table 1). It is rare to obtain Stage II CAPN5-NIV vitreous samples as the indication for vitrectomy in these patients is exceptional (e.g. in the case of epiretinal membrane formation, recalcitrant macular edema, or non-clearing vitreous opacities) as most symptoms in this stage can be managed without surgical intervention. Nevertheless, these data and analyses are valuable as they provide insight into underlying disease mechanisms in a rare patient population with few therapeutic options. Further prospective validation of these candidate biomarkers is required. Another limitation is the discovery-oriented nature of our proteomic analysis. Due to the rarity of surgical samples from CAPN5-NIV patients, we opted for an unbiased data-independent acquisition (DIA) approach which allows for the relative quantification of proteins in our samples. For more reliable and sensitive quantification of candidate biomarkers, a selective reaction monitoring (SRM) approach or multiplex ELISA would be more appropriate in future studies^11^.

## Conclusion

Proteomic analysis is a powerful tool for studying the molecular basis of inflammatory diseases, like CAPN5-NIV^9,11,43,44^. Although several CAPN5-NIV-causing mutations have been identified, it is unclear how a hyperactive protease leads to retinal disease. Thus, in the near-term the genotype of our CAPN5-NIV patient does little to change treatment outcomes. On the other hand, our proteomics dataset, points to differential expression of multiple disease-associated molecular pathways, a finding that has implications for the immediate treatment of CAPN5-NIV. Further validation of these novel biomarkers and drug targets will improve our understanding of molecular mechanisms underlying CAPN5-NIV and potentially point to therapies for more common causes of blindness.

## Methods

### Study approval

The study was approved by Stanford University and the University of Iowa’s Institutional Review Board and adhered to the tenets set forth in the Declaration of Helsinki (IRB: 201803853). Patients provided written informed consent for the use of their tissue samples. Patients underwent eye exams that included slit-lamp examination, dilated retinal bio-microscopy and indirect ophthalmoscopy. Data was collected from August 2009 to July 2018. Written informed consent was provided for pictures appearing in the manuscript.

### Sample collection

Pars plana vitrectomy was performed using a single-step transconjunctival 23-gauge trocar cannular system (Alcon Laboratories Inc, Fort Worth, TX), and an undiluted 0.5-cc sample of the vitreous was manually aspirated into a 3-cc syringe. Vitreous samples were immediately centrifuged in the operating room at 15,000 g for 5 minutes at room temperature to remove impurities and then finally stored at −80 °C, as previously described^9^.

### Proteomic analysis

Shotgun proteomic mass spectrometry-based measurements were performed in duplicate for control and CAPN5-NIV vitreous samples (Table 1). A liquid chromatography-tandem mass spectrometry (LC-MS/MS) approach was used for the relative quantitation and simultaneous identification of proteins from all samples. Briefly, vitreous protein concentration was determined using the Qubit Protein Quantification assay (Thermo Fisher). Proteins were then extracted from vitreous (20 µg protein per sample), precipitated in chloroform-methanol, dissolved in 0.1% Rapigest detergent in 50 mM ammonium bicarbonate. Trypsin was added to each sample at a ratio 1:40 enzyme/protein and digested overnight at 37 °C. The reaction was quenched by adding 90% formic acid to a final concentration of 2%. A total of 5 µl (1 µg of trypsin-digested peptides) of each sample was then injected into a ChromXP C18 reverse phase analytical column (10 cm in length). High-performance liquid chromatography (HPLC) was performed on a NanoLC Eskigent HPLC pump at a flow rate of 0.2 µl/min using a linear gradient of Buffer B (0.1% formic acid, 98% acetonitrile). Buffer A consisted of 5% acetonitrile, 0.1% formic acid. Mass spectrometry was performed on a Q Exactive^TM^ HF Hybrid Quadrupole-Orbitrap (Thermo Fisher) mass spectrometer equipped with a nano-LC electrospray ionization source (ThermoFinnigan). Full MS data were recorded on peptides over a 400 to 100 m/z range (positive ion mode). Data-independent acquisition (DIA) was used to generate MS data within a 25 Da fixed window (at 26% normalized collision energy). Biognoysis Spectronaut^TM^ Pulsar was used to search the DIA data. The human Uniprot database was used in the database search. Positive identification was set at 1% peptide FDR. K-Nearest Neighbor (KNN) imputation was for missing values. The mass spectrometry proteomics data have been deposited to the ProteomeXchange Consortium via the PRIDE partner repository with the dataset identifier PXD011987^45–47^.

### Statistical and bioinformatics analysis

Results were also saved in Excel as.txt format and were uploaded into the Partek Genomics Suite 6.5 software package. The data was normalized to log base 2 and compared using principal component and 1-way ANOVA analysis. For better interpretation, all proteins with non or less significant (p > 0.05) changes were eliminated from the table. The significant values were mapped using the ‘cluster based on significant genes’ visualization function with the standardization option chosen. Gene ontology (GO) analysis was performed in PANTHER^48,49^. Pie charts were created for the visualization of GO distributions within the list of proteins under the Batch ID search menu. Pie charts were created for each GO term category including biological process, molecular function, and cellular component. Reactome Pathway Analysis software was utilized to determine the most significant pathways^50^.

## Supplementary information

Supplemental Figures and Tables

## Data Availability

The mass spectrometry proteomics data have been deposited to the ProteomeXchange Consortium via the PRIDE partner repository with the dataset identifier PXD011987.
