# Supplementary material for: Proteomic insight into the pathogenesis of CAPN5-vitreoretinopathy
Source: Sci Rep. 2019 May 20;9:7608. doi: 10.1038/s41598-019-44031-7 (PMC6527583; doi:10.1038/s41598-019-44031-7)
Supplement: Supplementary file 1 — Supplemental Figures and Tables [file 41598_2019_44031_MOESM1_ESM.docx]

**Supplementary Online Content**

**Proteomic insight into the pathogenesis of CAPN5-vitreoretinopathy**

Gabriel Velez, Jing Yang, Angela S. Li, Stephen H. Tsang, Alexander G. Bassuk, Vinit B. Mahajan

**Table of Contents**

**Supplemental Figure 1.** CAPN5-NIV pedigree.

**Supplemental Table 1.** Early CAPN5-NIV pathways.

**Supplemental Table 2.** Late CAPN5-NIV pathways.

**Supplemental Figure 1. CAPN5-NIV pedigree.** CAPN5-NIV patients 2-5 belong to the same pedigree and are denoted by the orange circle. The remaining CAPN5-NIV vitreous samples are from unrelated patients (Supplemental Table 1).

**Supplemental Table 1. Early CAPN5-NIV pathways.**

| **Pathway name** | **p-value** | **FDR** |
| --- | --- | --- |
| Classical antibody-mediated complement activation | 1.1102230246251565E-16 | 1.9984014443252818E-15 |
| CD22 mediated BCR regulation | 1.1102230246251565E-16 | 1.9984014443252818E-15 |
| Scavenging of heme from plasma | 1.1102230246251565E-16 | 1.9984014443252818E-15 |
| Antigen activates B Cell Receptor (BCR) leading to generation of second messengers | 1.1102230246251565E-16 | 1.9984014443252818E-15 |
| Binding and Uptake of Ligands by Scavenger Receptors | 1.1102230246251565E-16 | 1.9984014443252818E-15 |
| Complement cascade | 1.1102230246251565E-16 | 1.9984014443252818E-15 |
| Regulation of Complement cascade | 1.1102230246251565E-16 | 1.9984014443252818E-15 |
| Creation of C4 and C2 activators | 3.3306690738754696E-16 | 5.329070518200751E-15 |
| Initial triggering of complement | 8.881784197001252E-16 | 1.2434497875801753E-14 |
| Hemostasis | 7.549516567451064E-15 | 7.993605777301127E-14 |
| Cell surface interactions at the vascular wall | 7.882583474838611E-15 | 7.993605777301127E-14 |
| FCGR activation | 7.993605777301127E-15 | 7.993605777301127E-14 |
| Role of LAT2/NTAL/LAB on calcium mobilization | 8.992806499463768E-15 | 8.992806499463768E-14 |
| Role of phospholipids in phagocytosis | 3.6415315207705135E-14 | 3.277378368693462E-13 |
| FCERI mediated Ca+2 mobilization | 5.051514762044462E-14 | 4.04121180963557E-13 |
| FCERI mediated MAPK activation | 5.617728504603292E-14 | 4.4941828036826337E-13 |
| Signaling by the B Cell Receptor (BCR) | 3.956834859764058E-13 | 2.7697844018348405E-12 |
| Regulation of actin dynamics for phagocytic cup formation | 1.1231016117108084E-12 | 7.861711281975658E-12 |
| FCERI mediated NF-kB activation | 4.244937734654286E-12 | 2.5469626407925716E-11 |
| Fcgamma receptor (FCGR) dependent phagocytosis | 7.560396753092391E-12 | 4.5362380518554346E-11 |
| Fc epsilon receptor (FCERI) signaling | 1.0520206927822073E-10 | 6.312124156693244E-10 |
| Innate Immune System | 3.7424818799536297E-10 | 1.871240939976815E-9 |
| Formation of Fibrin Clot (Clotting Cascade) | 1.9099120063259534E-9 | 9.549560031629767E-9 |
| Common Pathway of Fibrin Clot Formation | 2.482867467179517E-9 | 1.2414337335897585E-8 |
| Immunoregulatory interactions between a Lymphoid and a non-Lymphoid cell | 4.5511349133775525E-9 | 2.2755674566887762E-8 |
| Terminal pathway of complement | 1.1854425607804586E-7 | 5.927212803902293E-7 |
| Vesicle-mediated transport | 2.727978932748343E-7 | 1.0911915730993371E-6 |
| p130Cas linkage to MAPK signaling for integrins | 1.42514278189676E-6 | 5.70057112758704E-6 |
| GRB2:SOS provides linkage to MAPK signaling for Integrins | 1.42514278189676E-6 | 5.70057112758704E-6 |
| Immune System | 2.2049557472092474E-6 | 8.81982298883699E-6 |
| Platelet degranulation | 5.439691682651571E-6 | 1.6319075047954712E-5 |
| Response to elevated platelet cytosolic Ca2+ | 6.968838099141728E-6 | 2.0906514297425183E-5 |
| Integrin alphaIIb beta3 signaling | 1.6436526785734706E-5 | 4.930958035720412E-5 |
| Integrin signaling | 1.6436526785734706E-5 | 4.930958035720412E-5 |
| Signaling by high-kinase activity BRAF mutants | 4.35180183960826E-5 | 1.305540551882478E-4 |
| Platelet Aggregation (Plug Formation) | 6.528954766149564E-5 | 1.9586864298448692E-4 |
| MAP2K and MAPK activation | 6.528954766149564E-5 | 1.9586864298448692E-4 |
| Signaling by moderate kinase activity BRAF mutants | 1.0253880957178385E-4 | 3.0761642871535155E-4 |
| Paradoxical activation of RAF signaling by kinase inactive BRAF | 1.0253880957178385E-4 | 3.0761642871535155E-4 |
| Regulation of TLR by endogenous ligand | 1.5134984609643798E-4 | 4.5404953828931394E-4 |
| Intrinsic Pathway of Fibrin Clot Formation | 2.3231770641896077E-4 | 4.6463541283792154E-4 |
| Signaling by RAS mutants | 2.688437505932173E-4 | 5.376875011864346E-4 |
| Post-translational protein phosphorylation | 2.692083957863689E-4 | 5.384167915727378E-4 |
| Adaptive Immune System | 3.391174325509949E-4 | 6.782348651019898E-4 |
| Signaling by BRAF and RAF fusions | 4.125462696138449E-4 | 8.250925392276898E-4 |
| Platelet activation, signaling and aggregation | 4.7260205521326704E-4 | 9.452041104265341E-4 |
| Regulation of Insulin-like Growth Factor (IGF) transport and uptake by Insulin-like Growth Factor Binding Proteins (IGFBPs) | 5.254531853363664E-4 | 0.0010509063706727328 |
| Oncogenic MAPK signaling | 9.341894645468196E-4 | 0.0018683789290936392 |
| Integrin cell surface interactions | 0.001115184574945749 | 0.002230369149891498 |
| RAF/MAP kinase cascade | 0.012157210311013356 | 0.02431442062202671 |
| MAPK1/MAPK3 signaling | 0.0135087324406723 | 0.0270174648813446 |
| Extracellular matrix organization | 0.021802107584763752 | 0.043604215169527505 |
| MAPK family signaling cascades | 0.022922268161670423 | 0.045844536323340845 |
| VLDL assembly | 0.026209053152723483 | 0.05174253368587278 |
| Fibronectin matrix formation | 0.03136924096510174 | 0.05174253368587278 |
| VLDL clearance | 0.03136924096510174 | 0.05174253368587278 |
| Defective B4GALT1 causes B4GALT1-CDG (CDG-2d) | 0.04160910476597435 | 0.05174253368587278 |
| Defective CHST6 causes MCDC1 | 0.04160910476597435 | 0.05174253368587278 |
| Defective ST3GAL3 causes MCT12 and EIEE15 | 0.04160910476597435 | 0.05174253368587278 |
| Transport of gamma-carboxylated protein precursors from the endoplasmic reticulum to the Golgi apparatus | 0.04668905536496393 | 0.05174253368587278 |

**Supplemental Table 2. Late CAPN5-NIV pathways.**

| **Pathway name** | **p-value** | **FDR** |
| --- | --- | --- |
| CD22 mediated BCR regulation | 1.1102230246251565E-16 | 2.6645352591003757E-15 |
| Scavenging of heme from plasma | 1.1102230246251565E-16 | 2.6645352591003757E-15 |
| Complement cascade | 1.1102230246251565E-16 | 2.6645352591003757E-15 |
| Binding and Uptake of Ligands by Scavenger Receptors | 1.1102230246251565E-16 | 2.6645352591003757E-15 |
| Regulation of Complement cascade | 1.1102230246251565E-16 | 2.6645352591003757E-15 |
| Classical antibody-mediated complement activation | 3.3306690738754696E-16 | 5.662137425588298E-15 |
| Antigen activates B Cell Receptor (BCR) leading to generation of second messengers | 3.3306690738754696E-16 | 5.662137425588298E-15 |
| FCGR activation | 8.881784197001252E-16 | 1.1546319456101628E-14 |
| Hemostasis | 8.881784197001252E-16 | 1.1546319456101628E-14 |
| Creation of C4 and C2 activators | 1.2212453270876722E-15 | 1.4654943925052066E-14 |
| Initial triggering of complement | 3.552713678800501E-15 | 3.907985046680551E-14 |
| Role of phospholipids in phagocytosis | 5.218048215738236E-15 | 5.218048215738236E-14 |
| Cell surface interactions at the vascular wall | 1.0658141036401503E-14 | 9.592326932761353E-14 |
| Role of LAT2/NTAL/LAB on calcium mobilization | 2.5868196473766147E-14 | 2.0694557179012918E-13 |
| FCERI mediated Ca+2 mobilization | 1.6231460620019789E-13 | 1.2729817200352045E-12 |
| FCERI mediated MAPK activation | 1.8185453143360064E-13 | 1.2729817200352045E-12 |
| Regulation of actin dynamics for phagocytic cup formation | 2.6212365611399946E-13 | 1.8348655927979962E-12 |
| Fcgamma receptor (FCGR) dependent phagocytosis | 2.3047119768193625E-12 | 1.3828271860916175E-11 |
| Signaling by the B Cell Receptor (BCR) | 2.3047119768193625E-12 | 1.3828271860916175E-11 |
| Formation of Fibrin Clot (Clotting Cascade) | 1.499667057203169E-11 | 8.940070905794073E-11 |
| FCERI mediated NF-kB activation | 1.7880141811588146E-11 | 8.940070905794073E-11 |
| Terminal pathway of complement | 4.0019210167940855E-11 | 2.0009605083970428E-10 |
| Innate Immune System | 2.587139391607707E-10 | 1.2935696958038534E-9 |
| Common Pathway of Fibrin Clot Formation | 3.3420610723311484E-10 | 1.6710305361655742E-9 |
| Fc epsilon receptor (FCERI) signaling | 5.284843673791784E-10 | 2.1139374695167135E-9 |
| Immunoregulatory interactions between a Lymphoid and a non-Lymphoid cell | 2.727050829598454E-8 | 1.0908203318393817E-7 |
| Platelet degranulation | 3.96832967708427E-7 | 1.587331870833708E-6 |
| Response to elevated platelet cytosolic Ca2+ | 5.443294248674846E-7 | 2.1773176994699384E-6 |
| Intrinsic Pathway of Fibrin Clot Formation | 6.468133604276005E-7 | 2.587253441710402E-6 |
| Vesicle-mediated transport | 1.4800057505093278E-6 | 5.920023002037311E-6 |
| Platelet activation, signaling and aggregation | 1.9413375237697927E-5 | 5.824012571309378E-5 |
| Regulation of Insulin-like Growth Factor (IGF) transport and uptake by Insulin-like Growth Factor Binding Proteins (IGFBPs) | 3.362242837745555E-5 | 1.0086728513236665E-4 |
| Immune System | 9.611575957857443E-5 | 2.883472787357233E-4 |
| Post-translational protein phosphorylation | 1.3038219403760376E-4 | 3.911465821128113E-4 |
| p130Cas linkage to MAPK signaling for integrins | 1.870683588618416E-4 | 5.612050765855248E-4 |
| GRB2:SOS provides linkage to MAPK signaling for Integrins | 1.870683588618416E-4 | 5.612050765855248E-4 |
| Platelet Aggregation (Plug Formation) | 2.1365190242850218E-4 | 6.409557072855065E-4 |
| Regulation of TLR by endogenous ligand | 3.723919594634806E-4 | 0.0011171758783904417 |
| Integrin alphaIIb beta3 signaling | 0.001137706799637761 | 0.002275413599275522 |
| Integrin signaling | 0.001137706799637761 | 0.002275413599275522 |
| Transport of gamma-carboxylated protein precursors from the endoplasmic reticulum to the Golgi apparatus | 0.00198834827587524 | 0.00397669655175048 |
| Signaling by high-kinase activity BRAF mutants | 0.0023204245684754765 | 0.004640849136950953 |
| Removal of aminoterminal propeptides from gamma-carboxylated proteins | 0.002443345022238974 | 0.004886690044477948 |
| Gamma-carboxylation of protein precursors | 0.002443345022238974 | 0.004886690044477948 |
| Adaptive Immune System | 0.002789918240310385 | 0.00557983648062077 |
| Gamma-carboxylation, transport, and amino-terminal cleavage of proteins | 0.0029427176497284213 | 0.005885435299456843 |
| MAP2K and MAPK activation | 0.003118226246637934 | 0.006236452493275868 |
| Dissolution of Fibrin Clot | 0.004072020791002773 | 0.008144041582005546 |
| Signaling by moderate kinase activity BRAF mutants | 0.0043272782311973845 | 0.008654556462394769 |
| Paradoxical activation of RAF signaling by kinase inactive BRAF | 0.0043272782311973845 | 0.008654556462394769 |
| Signaling by RAS mutants | 0.00866860807710379 | 0.01733721615420758 |
| Signaling by BRAF and RAF fusions | 0.011772955948263664 | 0.023545911896527327 |
| Oncogenic MAPK signaling | 0.021001735337695338 | 0.042003470675390675 |
| Integrin cell surface interactions | 0.02378203462168338 | 0.04756406924336676 |
| Gamma carboxylation, hypusine formation and arylsulfatase activation | 0.032503293251623044 | 0.06500658650324609 |
| Retinoid metabolism and transport | 0.04043566377992969 | 0.06636338521290464 |
| Metabolism of fat-soluble vitamins | 0.04725095897761644 | 0.06636338521290464 |
